# Attentional Profiles and White Matter Correlates in Attention-Deficit/Hyperactivity Disorder Predominantly Inattentive Type

**DOI:** 10.3389/fpsyt.2015.00122

**Published:** 2015-09-15

**Authors:** Adriana Suzart Ungaretti Rossi, Luciana Monteiro de Moura, Claudia Berlim de Mello, Altay Alves Lino de Souza, Mauro Muszkat, Orlando Francisco Amodeo Bueno

**Affiliations:** ^1^Psychobiology Department, Universidade Federal de São Paulo, São Paulo, Brazil; ^2^Psychiatry Department, Universidade Federal de São Paulo, São Paulo, Brazil

**Keywords:** ADHD, inattentive type, attention, CPT, white matter, fractional anisotropy

## Abstract

Attention-deficit/hyperactivity disorder (ADHD) is a widely studied neurodevelopmental disorder. It is a highly heterogeneous condition, encompassing different types of expression. The predominantly inattentive type is the most prevalent and the most stable over the lifetime, yet it is the least-studied presentation. To increase understanding of its cognitive profile, 29 children with attention-deficit/hyperactivity disorder of predominantly inattentive type (ADHD-I) and 29 matched controls, aged 7–15 years, had their attentional abilities assessed through the Conners’ continuous performance test. Diffusion tensor imaging data were collected for all of the participants using a 3.0-T MRI system. Fractional anisotropy (FA) values were obtained for 20 fiber tracts, and brain-behavior correlations were calculated for 42 of the children. The ADHD-I children differed significantly from the typically developing (TD) children with respect to attentional measures, such as the ability to maintain response-time consistency throughout the task (Hit RT SE and Variability), vigilance (Hit RT ISI and Hit RT ISI SE), processing speed (Hit RT), selective attention (Omissions), sustained attention (Hit RT Block Change), error profile (Response Style), and inhibitory control (Perseverations). Evidence of significant differences between the ADHD-I and the TD participants was not found with respect to the mean FA values in the fiber tracts analyzed. Moderate and strong correlations between performance on the attention indicators and the tract-average FA values were found for the ADHD-I group. Our results contribute to a better characterization of the attentional profile of ADHD-I individuals and suggest that in children and adolescents with ADHD-I, attentional performance is mainly associated with the white matter structure of the long associative fibers that connect anterior–posterior brain areas.

## Introduction

Many studies have aimed to investigate cognitive and behavioral differences among the attention-deficit/hyperactivity disorder (ADHD) types, mainly between the combined (ADHD-C) and inattentive (ADHD-I) presentations (1–4). Although there is some divergence among the findings, significant differences in neuropsychological performance seem to reaffirm the differences among the types. ADHD-C individuals exhibit poorer inhibitory control, higher response variability (5), and higher behavior impulsivity (6). ADHD-I has been related to worse performance on cognitive tempo tests, with individuals appearing to orient and respond to cognitive and social stimuli slowly (6).

Therefore, the findings suggest that ADHD-I and ADHD-C individuals have different patterns of attentional deficits. The continuous performance test (CPT) is the most common neuropsychological instrument used for the evaluation of attention skills, and its indicators have been shown to be sensitive to the identification of attentional deficits in ADHD (7, 8). Different CPT versions are available, but the Conners’ continuous performance test II (CCPT-II) is the most popular version (9–11), and thus was utilized in the present study. Several studies focused on evaluating the performance of ADHD individuals on the CCPT-II have been conducted (12), but only a small number of the studies have compared different manifestations of the disorder (13–17). Among them, only one of the studies considered all of the indicators generated by the test in the analysis (14), which indicates a need for more studies to better characterize the ADHD types with respect to other attentional subcomponents, as measured by the CCPT-II indicators.

In addition to differences in attentional patterns, ADHD-C and ADHD-I also differ with respect to the age of onset and referral. Symptoms frequently occur several years later for ADHD-I (18, 19). Although ADHD-I is the most common type in population, ADHD-C are more likely to be referred for clinical services, suggesting that ADHD-I might be under-recognized and under-treated (20). Furthermore, so far, the majority of neuropsychological studies have been conducted with the combined type. Thus, considering that the inattentive type is the most prevalent manifestation of the disorder, presents more temporal stability throughout the course of development (5), and paradoxically has received the least attention to date, it is important to study individuals with this manifestation of the disorder, to better understand their cognitive particularities.

Developmental, cognitive, and behavioral differences between the types mentioned above confirm the clinical validity of the types, thereby suggesting the existence of phenotypic manifestations that, in turn, indicates possible underlying neurobiological differences (21).

Attention-deficit/hyperactivity disorders precise etiology is still unclear, but many structural and functional neuroimaging studies have investigated brain alterations (22) and have found statistically significant results. Nonetheless, the reported findings are still inconsistent (15, 23, 24). To date, the majority of neuroimaging studies have been conducted by clustering individuals with different presentations in the ADHD group, without considering the specific presentation of the disorder in their analysis. Precisely because of this point, a structural and functional neuroimaging review of ADHD (22) highlighted the need to consider the different manifestations of the disorder in future studies. Willcutt et al. (5) also affirmed that the lack of adequately powered brain imaging studies represents an important gap in the knowledge base regarding ADHD dimensions and types.

First, structural and functional studies investigated specific regions of abnormalities and dysfunction in ADHD (25). More recently, studies have exhibited a shift in perspective regarding the pathophysiology of the disorder, i.e., the focus has shifted from dysfunction on particular brain regions to emphasize alterations in distributed network organization (26). The adoption of diffusion tensor imaging (DTI) in pathophysiology studies is in accordance with this perspective shift, and as a non-invasive and non-ionizing magnetic resonance imaging technique, DTI is a method capable of mapping *in vivo* the white matter fiber architecture (24, 26, 27). DTI can estimates the directional diffusion of water molecules along axonal pathways through different measures: mean diffusivity (MD), which provides a measure of average diffusivity, axial diffusivity (AD), which describes the water mobility parallel to the axonal fiber, radial diffusivity (RD), which reflects water mobility perpendicular to axonal fiber and, finally, fractional anisotropy (FA), which express the “directionality” of diffusion in each voxel (27–29). Since FA values represent the amount of hindrance/restriction experienced by water molecules along the direction of white fiber and, thus, are a sensitive measure of changes in the white matter microstructure, they will be adopted here (27, 30, 31).

Usually, lower FA values are associated to factors that produce fewer barriers in a given space, and furthermore allow water molecules to diffuse more freely (e.g., larger axon diameter or lower packing density) or to increased membrane permeability (28, 32). Higher FA values might be a result of increased axonal packing, more myelination, higher fiber diameter or a less coherent organization of fibers’ axons (28). Indeed, the sensitivity of DTI to microstructural tissue characteristics makes this technique – and its measures, as FA – a powerful tool to investigate neurobiological underpins of several experimental and clinical conditions.

To the best of our knowledge, to date, only two recent DTI studies have considered ADHD types in their analysis (33, 34). Indeed, despite more than 20 years of neuroimaging research, relatively little is known about the neurobiology and etiology of the inattentive type (6), which justifies efforts to investigate the neurobiological characteristics of ADHD-I individuals.

Alterations in neural circuits that connect regions of the prefrontal cortex and the striatum have been associated with cognitive control deficits, which have been suggested to account for the inattention symptoms in ADHD (35–37). Nonetheless, according to Fernández-Perrone et al. (38), further studies that target a more comprehensive understanding of the symptomology of inattentiveness in ADHD and integrate findings from psychology, neurophysiology, neuroimaging, and other domains are required.

A few studies have already searched for associations between white matter fiber structure and attentional functioning, as measured by the CPT (39–42). Again, only one of those studies considered different presentations of the disorder in the analysis; however, correlations between white matter structure and performance on the CPT indicators were assessed strictly in the ADHD-C participants (25). Moreover, in this same study, only three CPT indicators were considered (Omissions, Commissions, and response Variability). Therefore, the possible associations between attentional measures, as measured by the CPT, and white matter structure in the inattentive type remain unexplored.

Thus, the present study aimed to compare individuals with ADHD-I and typically developing (TD) individuals with respect to attentional functioning as well as with respect to white matter measures (FA) to contribute to the characterization of individuals with this type. We also intended to investigate possible correlations between the attentional variables (CCPT) and the white matter measures (FA).

## Materials and Methods

### Participants

The sample of the present study was composed of 29 ADHD-I children, aged 7–15 years, and 29 TD children matched for age, sex, and type of school (public or private). Participants were recruited using a purposive sampling method, from 2010 to 2014.

The ADHD-I participants were referred from public and private centers, where they had their diagnosis made by an ADHD clinician expert (neuropaediatrician or child and adolescent psychiatrist) based on the DSM-IV parameters. The ADHD individuals must have presented six or more inattention symptoms and up to five hyperactivity/impulsivity symptoms to be classified as with the inattentive type. All of the TD children were enrolled from the community.

The exclusion criteria included an estimated intelligence quotient (IQ) of 85 or lower, neurological disease, sensory deficits, or another condition that would prevent participation in the procedures.

Only three of the ADHD-I children were using methyl- phenidate, and the use was for a few months (2, 5, and 13 months) until assessment time. They were without medication at least 24 h before testing.

Seven children from the TD group and nine children from the ADHD-I group were excluded from imaging analysis due to excessive motion during imaging or to artifacts. Thus, 22 TD and 20 ADHD-I images met the quality criteria for processing and comprised this stage of the analysis. Only two of the three children who were using methylphenidate had their images analyzed due to quality criteria.

This study was approved by the Ethics Committee of the Federal University of São Paulo. The parents provided written informed consent for all of the participants. The children provided verbal or written informed assent.

### Instruments

All of the subjects were subjected to a neuropsychological protocol, as described here. To identify the presence of possible exclusion criteria, the level of intellectual functioning was assessed through the Block Design and Vocabulary subtests of the abbreviated version of the Wechsler intelligence scale for children, third edition (WISC-III) (43). Both subtests have been shown to be valid measures for the estimation of total IQ in TD children as well as in ADHD children (44).

All of the participants were subjected to a neuropsychological evaluation, which included an examination of the subject’s intellectual level, a computerized attention test, and tests of working memory, visual constructive functions and visual memory, verbal fluency, decision making, facial emotion recognition, and academic performance. This evaluation was used to exclude other diagnostic conditions.

Attention was evaluated through the response to the standard version of the CCPT-II (9). Twelve indicators of the CCPT-II were selected to compare the ADHD-I children to the TD children with respect to attentional functioning. The selected indicators were Omissions, Commissions, Hit Reaction Time (Hit RT), Hit Reaction Time Standard Error (Hit RT SE), Variability, Detectability, Response Style, Perseverations, Hit Reaction Time Block Change (Hit RT BC), Hit Reaction Time Block Change Standard Error (Hit RT BC SE), Hit Reaction Time Inter-stimulus Interval (Hit RT ISI), and Hit Reaction Time Inter-stimulus Interval Standard Error (Hit RT ISI SE).

### DTI image acquisition protocol

A 2D MR-DTI sequence (TR = 6500 ms, TE = 95 ms, flip angle = 90°, matrix size = 128 × 128, NEX = 1, FOV = 230 mm, 12 directions, *b* = 1000 m/s^2^, thickness = 4 mm with the gap between slices = 0.8 mm, yielding 30 slices) was acquired using a 3.0-T, 43 mT/m gradient MR system (Magnetom Verio, Siemens Medical Systems, Erlangen, Germany) in all of the subjects. All of the images were checked for quality, which was rated based on an initial visual inspection. This procedure was followed by inspection for and the detection of artifacts, such as geometrical distortions, susceptibility effects, and motion.

### DTI image post-processing

Nifti images were processed using the FSL platform (45), following these steps: correction of eddy currents and skull extraction using the BET tool. An FDT tool was then used to generate FA maps. All of the FA maps were then merged in a 4D image using tract-based spatial statistics (TBSS) (45). Because the sample comprised children, all of the FA images were aligned to identify the most representative FA map to use as a reference image, flag-n. At least a mean FA skeleton was generated, and the FA values of the most relevant tracts from the spatially normalized FA map of each subject were then projected onto this skeleton using a threshold of 0.3. Permutation-based non-parametric inference was applied to the unsmoothed statistical maps using 10,000 permutations, and the cluster-like structures were enhanced using the threshold-free cluster enhancement (TFCE) algorithm (46) at a *p* level <0.05 (FWE correction).

We then used the 4D skeletonized FA image, with all of the projected mean skeletons of each subject, and the skeleton mask of the group to run an automated region of interest (ROI) extraction, using R project 3.0.3. The extraction of the tracts was based on the 20 tracts of the JHU (John Hopkins University) DTI white matter tractography atlas (47, 48), resulting in a mean FA value for each tract of each subject.

### Statistical analysis

The demographic characteristics of the samples were statistically analyzed using Student’s *t*-test and chi-square tests (statistical software SPSS version 19.0). To minimize the age effect, the raw neuropsychological measures were standardized into *z*-score measures, adopting the Brazilian population norms (49, 50). Because the neuropsychological measures did not exhibit a normal distribution, they were converted into *z*-scores. *T*-tests and Cohen’s *d* effect size were adopted to compare the performance of the groups on the cognitive measures.

Because a normal distribution was also not found for the FA values of the fiber tracts, these values were also transformed into *z*-scores, and group comparison was made using *T*-tests. Cohen’s *d* effect size was also calculated. Correlations between the attentional measures and the FA values were assessed using Pearson’s correlation. A significance level of *p* ≤ 0.05 was adopted.

The power calculation was performed based on the collected sample size of 58 participants (using *post hoc* GPower in option 3). It was calculated the minimum effect size (based on Cohen’s *d*) for a 58 subjects sample (2 groups of 29) so that it would have the power to detect an 80% of difference expected in population. Statically significant differences were considered to have an observable effect in the population with 80% of power when the *d* value was >0.67 (i.e., the minimum effect size for this sample should be *d* = 0.67).

Main analyses were run including all subjects. For comparative purposes, neuroimaging analysis and correlations between attentional measures and FA values were run as well excluding children who were in use of methylphenidate.

## Results

Table 1 shows the mean age, gender frequency, and distribution of school type in the TD and ADHD-I groups. The groups did not differ significantly with respect to age, gender or type of school attended. Both of the groups comprise primarily by males, consistent with the higher prevalence of ADHD among males (20). There was a statistically significant difference between the groups with respect to estimated IQ (*F* = 0.23, df = 55, *p* = 0.02), but the mean estimation of intellectual functioning was above the average range in both groups. Based on the recommendations of Dennis et al. (51), the cognitive data were not controlled for IQ, and IQ was not used as a covariate.

**Table 1:** **Sociodemographic characteristics of groups**.

	Typically development group (*n* = 29)	ADHD-I group (*n* = 29)	Test statistic	*p*-Value
Demographic				
Age (years)	10.10 (1.63)	10.14 (1.94)	*t* = −0.73	0.94
Gender (% male)	65.5%	69.0%	χ^2^ = 0.78	0.78
Type of school (% public)	62.1%	50.0%	χ^2^ = 0.84	0.36
Estimated IQ	120.21 (14.96)	110.60 (14.94)	*t* = −3.26	0.02*

**t* = *T*-test; χ^2^ = Chi-square*.

*Significant at p < 0.05.

As shown in Table 2, the evaluation of attentional functioning indicated that, compared to the matched TD individuals, the ADHD-I subjects presented poorer performance on the following indicators: Omissions, Hit RT, Hit RT SE, Variability, Response Style, Perseverations, Hit RT Block Change, Hit RT ISI, and Hit RT ISI SE.

**Table 2:** **Comparison of the TD and ADHD-I groups with respect to performance on the CCPT indicators (*T*-test)**.

Attentional measure	TD (SD)	ADHD-I (SD)	*t*	*p*-Value	Cohen’s *d*
CCPT omissions	0.556 (0.421)	−0.058 (1.101)	−5.17	0.001**	0.736
CCPT commissions	0.119 (1.080)	−0.128 (0.913)	−0.92	0.363	0.247
CCPT hit RT	0.442 (0.812)	−0.458 (0.982)	−3.78	0.001**	0.998
CCPT hit RT SE	0.590 (0.363)	−0.634 (1.081)	−5.764	0.001**	1.518
CCPT variability	0.518 (0.431)	−0.556 (1.137)	−4.74	0.001**	1.249
CCPT detectability	0.144 (1.112)	−0.155 (0.857)	−1.12	0.267	0.301
CCPT response style	0.292 (0.455)	−0.314 (1.302)	−2.36	0.022*	0.621
CCPT perseverations	0.254 (0.118)	−0.273 (1.397)	−2.02	0.048*	0.532
CCPT hit RT block change	0.282 (0.622)	−0.292 (1.224)	−2.24	0.029*	0.591
CCPT hit RT block change SE	0.136 (0.899)	−0.146 (1.096)	−1.05	0.296	0.281
CCPT hit RT inter-stimulus interval	0.484 (0.761)	−0.520 (0.974)	−4.32	0.001**	1.149
CCPT hit RT inter-stimulus interval SE	0.448 (0.779)	−0.481 (0.998)	−3.89	0.001**	1.038

*ADHD-I, attention-deficit/hyperactivity disorder inattentive type; TD, typically developing; SE, standard error; CCPT, Conners’ continuous performance test II; RT, reaction time; *t*, *T-*test*.

*Significant at *p* < 0.05.

**Significant at *p* < 0.01.

Calculation of the effect sizes revealed that the groups were best discriminate – in order of discriminating strength – by Hit RT SE, Variability, Hit RT ISI, Hit RT ISI SE, Hit RT, Omissions, Hit RT Block Change, Response style, and Perseverations.

Hit RT Block Change, Response style, and Perseverations indicators were statistically different between groups but since their effect size (Cohen’s *d*) was under 0.67, the current sample size does not have the power to assume that these differences are observable in the population (based at 80% statistical power). Thus the differences regarding these indicators should be taken cautiously.

Neuroimaging processing was performed for 42 participants (22 TD and 20 ADHD-I subjects). First, a TFCE statistical analysis was run in TBSS to compare the ADHD-I group and the TD group. Differences were found between the groups at *p* < 0.05, but the findings were not significant after FWE correction. Thus, there was no evidence of a significant difference between the groups at the level of *p* < 0.05, FWE corrected.

Then, the ADHD-I and TD groups were compared with respect to the mean FA values of 20 tracts. The FA values were standardized as *z*-scores. As presented in Table 3, the results indicated that there was no evidence of a statistically significant difference between the groups with respect to the considered tracts.

**Table 3:** ***T*-tests comparing the *z*-scores of mean FA values for the TD and ADHD-I groups**.

FA mean value	TD (SD)	ADHD-I (SD)	*t*	*p*-Value	Cohen’s *d*
Anterior thalamic radiation L	0.098 (0.915)	−0.108 (1.099)	−0.66	0.509	0.208
Anterior thalamic radiation R	0.014 (0.988)	−0.015 (1.038)	−0.97	0.923	0.029
Corticospinal tract L	0.069 (0.930)	−0.076 (1.091)	−0.46	0.645	0.143
Corticospinal tract R	−0.221 (0.206)	−0.243 (1.412)	−1.52	0.136	0.022
Cingulum (cingulate gyrus) L	−0.115 (1.134)	0.127 (0.393)	0.78	0.441	0.285
Cingulum (cingulate gyrus) R	0.078 (0.829)	−0.086 (1.176)	−0.53	0.600	0.161
Cingulum (hippocampus) L	−0.125 (1.184)	0.137 (0.754)	0.88	0.402	0.264
Cingulum (hippocampus) R	0.724 (0.907)	−0.079 (1.114)	−0.48	0.628	0.791
Forceps major	0.024 (1.001)	−0.026 (1.024)	−0.16	0.873	0.049
Forceps minor	0.022 (0.994)	−0.024 (1.031)	−0.14	0.886	0.045
Inferior fronto-occipital fasciculus L	−0.168 (1.142)	0.184 (0.806)	1.14	0.426	0.356
Inferior fronto-occipital fasciculus R	0.031 (1.018)	−0.034 (1.005)	−0.21	0.836	0.064
Inferior longitudinal fasciculus L	−0.116 (1.063)	0.127 (0.935)	0.78	0.438	0.243
Inferior longitudinal fasciculus R	0.119 (0.297)	−0.131 (1.423)	−0.81	0.423	0.243
Superior longitudinal fasciculus L	0.023 (1.008)	−0.026 (1.017)	−0.16	0.876	0.048
Superior longitudinal fasciculus R	0.093 (0.909)	−0.103 (1.106)	−0.63	0.532	0.194
Uncinate fasciculus L	0.017 (1.011)	−0.019 (1.013)	−0.18	0.908	0.036
Uncinate fasciculus R	0.024 (0.995)	−0.026 (1.031)	−0.16	0.875	0.049
Superior longitudinal fasciculus (temporal part) L	−0.117 (1.091)	0.129 (0.898)	0.79	0.432	−0.246
Superior longitudinal fasciculus (temporal part) R	0.076 (0.952)	−0.083 (1.069)	−0.51	0.612	0.157

*L, left; R, right; ADHD-I, attention-deficit/hyperactivity disorder inattentive type; TD, typically developing; *t*, *T*-test*.

*Significant at *p* < 0.05.

**Significant at *p* < 0.01.

Comparative analysis excluding the two ADHD-I participants who were in use of methylphenidate were possible since groups remained not differing statistically in respect to mean age or sex distribution. Results revealed similar results (i.e., no evidence of statistically significant differences between groups).

Pearson’s correlations were calculated for the ADHD-I group to identify possible associations between attentional functioning (measured by performance on the CCPT indicators) and the neurobiological data (measured by the mean FA values of the 20 fiber tracts) in the individuals with ADHD-I. Standardized measures of attentional performance and the neuroimaging data were adopted in this analysis, and some of the correlations were found to be statistically significant for the ADHD-I group (Table 4). Correlations between performance on other CCPT indicators and the FA values of other fiber tracts were duly considered in this analysis but were not statistically significant and therefore are not shown in Table 4. With the exception of one negative correlation, all of the correlations found were positive. All of the correlations were moderate or strong.

**Table 4:** **Significant Pearson’s correlations between the FA values of the fiber tracts (*z*-score) and the CCPT indicators (*z*-score) in the ADHD-I group**.

Fiber tract	Omissions	Detectability	Response style	Perseverations	Hit RT BC	Hit RT BC SE	Hit RT ISI
ATR L	0.25	0.25	−0.11	−0.08	−0.09	−0.29	0.56*
CST L	0.07	−0.16	0.54*	−0.09	0.28	0.13	−0.21
CST R	−0.28	−0.47*	−0.26	−0.09	−0.20	−0.18	−0.78
CGH R	0.21	−0.12	−0.20	0.63**	−0.01	0.01	0.31
Forceps major	−0.11	−0.28	0.95**	−0.08	−0.04	−0.04	−0.05
Forceps minor	−0.19	0.05	−0.13	−0.09	0.66*	0.57*	−0.06
IFOF R	0.12	0.21	0.52*	0.50*	−0.29	−0.18	0.46*
UF L	0.50*	0.17	−0.06	−0.01	0.18	0.15	0.29
UF R	0.49*	0.08	−0.06	0.06	0.23	0.24	0.08

*CCPT, Conners’ continuous performance test II; ATR L, anterior left thalamic radiation; CST L, left corticospinal tract; CST R, right corticospinal tract; CGH R, right cingulum (hippocampus); IFOF R, right inferior fronto-occipital fasciculus; UF L, left uncinate fasciculus; UF R, right uncinate fasciculus; RT, reaction time; BC, block change; SE, standard error; ISI, inter-stimulus interval*.

*Significant at *p* < 0.05.

**Significant at *p* < 0.01.

*Bold font indicates significance either at p < 0.01 or 0.05*.

The very same Pearson’s correlations (comprising the same variables) were run excluding the two ADHD-I subjects who were in use of methylphenidate. The same results were found, with the exception of the correlation between the right corticospinal tract (CST R) and the Detectability indicator. Although CST R and Detectability correlation were not statistically significant for the analysis without the two medicated children, its *p*-value was 0.06, what indicates an almost significant tendency.

Taking into account that results were very much similar in the analyses with and without the medicated participants and that the time of use of the methylphenidate was short for both children (2 and 13 months), discussion will be conducted considering the findings of the analysis that comprised all participants.

## Discussion

### Attentional performance

The participants in the ADHD-I group showed significantly lower mean IQ scores than the participants in the TD group, but previous studies have concluded that ADHD children exhibit lower IQ scores when compared with their peers (52, 53), possibly due to attentional problems and suboptimal test-taking behavior rather than reduced intelligence. Furthermore, there are strong recommendations to not attempt to control for IQ differences using matching procedures or as is more common, using IQ score as a covariate. This recommendation was based, among other reasons, on the fact that IQ does not meet the requirements for a covariate; as a result, this procedure would produce overcorrected data, possibly introducing a bias to the findings (51).

With respect to attentional functioning, which was measured by performance on the CCPT indicators, the ADHD-I group showed worse performance than the TD group on the measures related to the ability to maintain a consistent response time throughout the task (Hit RT SE and Variability), vigilance (Hit RT ISI, Hit RT ISI SE), processing speed (Hit RT), selective attention (Omissions), sustained attention (Hit RT Block Change), and inhibitory control (Response Style and Perseverations).

The Hit RT SE and Variability indicators, as mentioned above, evaluate the ability to maintain a stable response time throughout the test. Intra-individual variability, as assessed by a variety of speed-based reaction-time (RT) tasks, is one of the most consistent manifestations among ADHD individuals. This “moment-to-moment” variability and inconsistency in performance is considered a core feature of the disorder (21, 54, 55). Consistent with our findings, a study that compared ADHD-I subjects to controls with respect to performance on the Variability indicator of the CCPT revealed that the ADHD-I group exhibited worse performance on this measure (13).

Our results also showed that the ADHD-I group presented lower scores on Hit RT ISI and Hit RT ISI SE, which are measures of vigilance. These measures evaluate the ability to adapt performance (or reaction time) in accordance to the different intervals in which stimuli are presented (1, 2, and 4 s). Thus, our results suggest that ADHD-I individuals have more difficult in rapidly adapt their performance based on changes in the environment. Among the previous studies that investigated the performance of ADHD individuals with different types on the CPT, Egeland et al. (15) and Rivero et al. (14) were the only ones to include the Hit RT ISI indicator. The results of Egeland et al. suggested that only the ADHD-C subjects performed worse than the controls on this measure (i.e., there was no significant difference between the ADHD-I and the control group on this measure). On the other hand, Rivero et al. (14) found that the ADHD-I subjects performed worse than the controls for both the Hit RT ISI and Hit RT ISI SE measures, suggesting that individuals with this presentation of the disorder also have vigilance deficits.

In addition to vigilance deficits, the ADHD-I participants exhibited slower processing speed as well. The ADHD-I group performed significantly worse than the TD group with respect to the Hit RT measure. This finding is in line with the results from previous studies (13, 14), in which the ADHD-I group presented slower reaction times than the controls. These results are in accordance with the previous results that affirmed that with respect to cognitive profile, individuals with the ADHD-I presentation could be differentiated mainly by worse performance on cognitive tempo tests, with slow orientation and responses to stimuli (6). Another study also argued that the deficits in processing speed were a neuropsychological characteristic of the “restrictive presentation,” but our sample was not divided into a restrictive inattentive and predominantly inattentive group and possibly included individuals with both presentations.

Studies that compared the performance of patients with ADHD on the CCPT to the performance of normal controls have frequently concluded that the number of Omissions is a measure that is impaired in ADHD individuals (13, 14, 39, 53, 56–58). Indeed, a meta-analysis that included different CPT versions concluded that the number of Omissions is one of the most impaired measures in individuals with the disorder (12), indicating a deficit in the ability to respond to target stimuli (i.e., selective attention). Impairment of this ability is highly associated with the inattention criteria of the disorder. Rizzutti et al. (13) also concluded that individuals with ADHD-I perform worse than controls and individuals with ADHD-C on this CCPT measure.

The Hit RT BC indicator is a measure of the change in reaction time to stimuli throughout the task. Impaired performance on this indicator would suggest slower reaction times across the test, and thus a deficit in sustaining attention. Our findings demonstrated worse performance of the ADHD-I individuals on this measure, and this result is in accordance with the findings of Egeland et al. (15). These authors also found that the ADHD-I group presented worse performance than healthy controls with respect to this measure.

Response style is an indicator that intends to identify the style of response during the test: either a more conservative or a more free-responding profile. The individuals with ADHD-I presented significantly higher scores on these measures, which suggest a tendency to respond in more cautious way, choosing to respond infrequently to guarantee that the responses given are correct. A previous study that compared ADHD-I, ADHD-C, and controls on this measure did not find significant differences (14, 16). Nonetheless, considering that our ADHD-I sample was characterized by more frequent omission errors and by the absence of a difference in Commission errors, it is understandable that the ratio of these kinds of errors suggests a more conservative profile, i.e., a profile with less commission than omission errors.

In our results, the ADHD-I group presented a higher frequency of perseverative (anticipated) responses than the TD group. This higher number of Perseverations is indicative of greater impulsiveness (49). Although the inattentive type is known for not presenting as high a level of impulsivity as the combined type, it is possible that ADHD-I individuals still have reasonably worse inhibitory control than TD individuals. Given that the effect size for this measure was the smallest among the considered variables, we can assume that perseveration may not be the most identifying characteristic of this type, but it happens often enough to differentiate individuals with this type from TD children. The results obtained by Rizzutti et al. (13) and Rivero et al. (14) corroborate this assumption because their findings indicated that the ADHD-I groups presented significantly more perseverative responses than the control groups, although they did not differ significantly from the ADHD-C individuals. Both the ADHD-I and the ADHD-C individuals presented statistically significantly worse performance than the controls.

### Whole-brain analysis in TBSS

The groups were compared with regard to the mean FA values of the 20 tracts, and the results revealed no evidence of significant differences between the ADHD-I and TD groups. To date, DTI studies of ADHD individuals have produced very variable results (23). A study that compared white matter measures in children and adolescents with ADHD, unaffected siblings of ADHD probands and healthy controls (*n* = 17) did not find evidence of statistically significant differences between the groups with respect to FA values. Widespread differences were obtained through MD and AD measures (30).

### White matter – behavior correlations

In the ADHD-I group, the attentional measures obtained through the CCPT performance were correlated with the mean FA values extracted of the ROIs of the 20 fiber tracts analyzed in this study. The majority of the correlations found were moderate and positive, suggesting that the better the performance on a given attentional subcomponent, the higher the FA value of certain fiber tracts. In addition, increased FA values may be related to more myelinated tracts or higher fiber density. The correlations found will now be briefly discussed.

The uncinate fasciculus (UF) is a hook-shaped bundle of fibers that bidirectionally connects the lower surfaces of the frontal lobe to structures in the anterior temporal lobe, such as the hippocampus and amygdala (59). Our findings indicated that a higher frequency of omission errors is associated with lower FA values in both the right and left UF. These findings are in line with the results of a study performed with adults with ADHD, which showed that greater inattention but not hyperactivity-impulsivity was associated with significantly lower FA values in the left UF (60).

The forceps minor is a commissural tract that connects the medial and lateral surfaces of the frontal lobes (61). The results of this study revealed that both the Hit RT BC and Hit RT BC SE indicators, which are measures that identify the possible augmentation of reaction time throughout the task, were moderately and positively correlated with the forceps minor. This finding suggests that the greater the consistency in reaction time, the higher the FA value of this tract. Similar findings were obtained by a study that correlated measures of intra-individual variability in reaction time measures with white matter and found that lower intra-individual variability (or higher reaction time consistency) was associated with higher FA values in the forceps minor (62).

The Hit RT ISI indicator, which is a measure of vigilance, evaluates the ability to change response speeds across the different inter-stimulus intervals of the CCPT. This indicator was positively and moderately associated with the FA values of the left anterior thalamic radiation (ATR) and the right inferior fronto-occipital fasciculus (IFOF). A previous study found that the majority of the processing speed clusters projected on the ATR and the forceps minor (63), highlighting the role of the ATR in processing speed. Considering that adequate performance on Hit RT ISI is highly dependent on the ability to discriminate and respond adequately to different speeds of stimulus presentation, it is possible to comprehend why this measure was associated with the measures of ATR structure. The IFOF, integrates the visual and auditory association cortices with the prefrontal cortex (61) and plays a role in attentional control (60, 64). The connections between occipital and frontal areas are very important for top-down processing, which allows individuals to adjust their reaction time in accordance with the speed of stimulus presentation. This relation contributes to understanding of the association found between this CCPT indicator and the right IFOF’s FA values.

The right IFOF was also moderately associated with a measure of perseveration, indicating that the higher the FA value, the less frequent the perseverative errors on the CCPT. As discussed previously, perseverations are errors that are influenced by impulsivity – or poor inhibitory control – and thus it is possible to infer that a higher FA of the IFOF, which is a tract associated with attentional control, may exert an expressive influence on the control of impulsive responses. This measure of perseverative errors was also positively associated with the hippocampal portion of the right cingulum (CGH). A previous study also identified that the integrity of the CGH bundle is associated, among other functions, with inhibition performance in both ADHD and control individuals, which is in line with our findings (42).

The FA values of the right IFOF, along with the left CST and the forceps major, were also associated with another CCPT measure, the Response Style indicator. According to our results, the FA values of the forceps major, the right IFOF and the left CS were associated with the ability to adequately discriminate between target and non-target stimuli. The forceps major consists of the splenium of the corpus callosum and the associated white matter radiations that interconnect the occipital lobes (65), thus effecting visual processing. Because this CCPT measure evaluates the ability to adequately distinguish between target and non-target stimuli, the association between this measure and this fiber tract is understandable. The IFOF also influences visuospatial processing (66) and, moreover, plays a role in attentional control (60, 64), allowing conscious decisions to respond or to inhibit a response to a given stimulus. As a tract associated with sensorimotor functions (61), the CST may exert complementary control on response inhibition.

Finally, detectability, which is a measure of the individual’s ability to distinguish and detect target from non-target stimuli, demonstrated a negative correlation with the right CST. Curiously, this measure was the only one to present a negative correlation with the FA values of a fiber tract, namely the right CST. On the other hand, the left CST was positively associated with the ability to inhibit motor responses to non-target stimuli (Response Style indicator). It is possible to assume that in the ADHD-I individuals, better stimulus discrimination indicates a reduced requirement for these motor fibers on the right side, which occurs through hemispheric compensation, with these functions taken on by the left CST.

## Conclusion

We can conclude that our findings suggest that compared to matched TD individuals, the ADHD-I attentional profile is characterized by more variable performance, difficulty in rapidly adapting performance in accordance with environmental requirements, slower processing speed, less efficient responses (higher number of Omissions), and reduced duration of the engagement of focus on tasks. In the ADHD-I group, attentional measures (e.g., selective attention, stimulus discriminability, the ability to adjust performance to environmental demands and inhibitory control) were associated with the associative, projection, and commissural tracts but mainly with the long associative fibers that connect posterior cortical and subcortical areas to the frontal lobe. These findings indicate that attention is a complex cognitive function that depends on the integration of multiple brain areas and, more intensively, on the adequate development of frontal regions. Previous studies also concluded that circuits that connect the prefrontal cortex and motor areas exert an important influence on the inattention deficits of ADHD individuals (35, 36).

This study contributes to a better delineation of the attentional profile of ADHD-I individuals and the association of their deficits with structural white matter particularities. We highlighted the extensive involvement of the long associative fibers that connect anterior–posterior brain areas, as the IFOF, in attentional functioning in children and adolescents with ADHD-I.

The limitations of this study include the use of attentional measures extracted from only one attentional test. Three of the participants in the ADHD-I group were using methylphenidate for approximately up to a year, which may have had a minimal impact on the brain microstructural properties. The small size of our sample prevented some generalization of our findings. Our sample size just allowed us to affirm that a finding would be representative enough to consider it as an observable effect in the population (at 80% of power) when it had an effect size greater than *d* = 0.067. Thus, part of the findings regarding the attentional measures could not be extrapolated to the population.

Future studies should replicate the present procedures, including ADHD-C individuals as a third group in addition to the ADHD-I and TD individuals, to allow individuals from different types to be compared with respect to the same variables. So far, the studies performed to investigate attentional performance associations with white matter structure in ADHD have either grouped different type individuals into the same group or selected one type of the disorder to better analyze, as done here. Nonetheless, comparisons across studies are limited because the methods and procedures are variable. Thus, we suggest that further studies utilize larger samples in their cognitive-brain structure analysis and include, in addition to TD individuals, the other presentations of the disorder, i.e., the restrictive inattention (proposed by the DSM V), inattentive, combined, and hyperactive–impulsive-presentations, to allow increased understanding of these different manifestations of the disorder.

## Conflict of Interest Statement

The authors declare that the research was conducted in the absence of any commercial or financial relationships that could be construed as a potential conflict of interest.
